# Prospective Pilot Study of Ultrasound Resolution Microscopy Imaging (URM) for Differentiating Benign and Malignant Breast Lesions: A Quantitative Microvascular Parameter Analysis

**DOI:** 10.3390/diagnostics16081119

**Published:** 2026-04-08

**Authors:** Fan Li, Nuo Xu, Jun Wu, Rui Hu, Zhi Chen, Ji’ao You, Xiaofeng Lan, Fang Ma, Xiang Xie

**Affiliations:** 1 Department of Ultrasound Medicine, The Second Affiliated Hospital of Anhui Medical University, Hefei 230601, China; fanl1318@126.com (F.L.); 13516454113@163.com (N.X.); wujun201866@163.com (J.W.); 15922437869@163.com (R.H.); shikou1123@163.com (Z.C.); youjiaojump@163.com (J.Y.);; 2 Department of Ultrasound Medicine, The Second People’s Hospital of Hefei, Hefei Hospital Affiliated to Anhui Medical University, Hefei 230011, China

**Keywords:** ultrasound resolution microscopy (URM), breast masses, quantitative parameters, prospective study, LASSO regression

## Abstract

**Objective:** Ultrasound Resolution Microscopy (URM) is an emerging technique that provides superior delineation of tumor microvasculature. This prospective study aimed to evaluate the diagnostic value of URM in differentiating benign from malignant breast lesions. **Methods:** From September 2024 to October 2025, 55 patients with 57 breast masses underwent conventional ultrasound and contrast-enhanced URM. Microvascular parameters were quantitatively analyzed and cross-referenced with histopathology. To mitigate overfitting, LASSO regression was employed to screen 14 URM indices. A combined predictive model integrating core URM features with BI-RADS categorization (dichotomized at 4A) was developed and evaluated using ROC and decision curve analysis (DCA). **Results:** Thirty-four malignant and 23 benign masses were confirmed. Malignant lesions exhibited comprehensively elevated microvascular abundance and architectural chaos. LASSO regression distilled these features down to two core independent predictors: Vessel Count and Max Curvature. The BI-RADS-alone model yielded 100% sensitivity but extremely low specificity (30.43%). Crucially, the Combined model significantly outperformed the single-modality approaches, achieving an excellent AUC of 0.896 (vs. 0.652 for BI-RADS alone, *p* < 0.001). By integrating URM parameters, the Combined model maintained adequate sensitivity (73.53%) while drastically boosting specificity to 91.30%. DCA confirmed superior net clinical benefit for the combined strategy. **Conclusions:** Quantitative URM imaging effectively characterizes the distinct microvascular features of breast cancers. Integrating URM functional parameters with conventional BI-RADS categorization significantly improves diagnostic specificity. Consequently, this combined approach provides a reliable non-invasive strategy to optimize risk stratification, effectively minimizing false-positive diagnoses and averting unnecessary invasive biopsies in routine clinical practice.

## 1. Introduction

Breast cancer remains the most frequently diagnosed malignancy and the leading cause of cancer-related mortality among women worldwide [1]. According to the latest epidemiological data from the World Health Organization (WHO) and the GLOBOCAN 2022 estimates, there were approximately 2.3 million new breast cancer cases and 670,000 associated deaths globally in 2022 [1]. Furthermore, the WHO and the International Agency for Research on Cancer (IARC) project a daunting forecast for the coming decades: driven by demographic changes and population aging, the global burden of breast cancer is estimated to surge by more than 40%, reaching over 3 million new cases annually by 2040 [2]. Confronted with this escalating public health challenge, although mortality rates have gradually declined in certain regions—largely attributed to advancements in screening, early detection, and improved treatment strategies [3]—the pressing need for highly accurate, non-invasive diagnostic modalities remains paramount to optimize patient triage and reduce unnecessary invasive procedures.

Tumor angiogenesis, a hallmark of cancer, plays a critical role in the development, progression, and metastasis of breast cancer [4,5]. The microvascular architecture of malignant breast lesions is typically characterized by chaotic distribution, irregular branching, and peripheral penetrating vessels, which differs significantly from the organized vasculature of benign entities [6,7]. Pathologically, microvessel density (MVD) serves as the gold standard for assessing tumor angiogenesis [8]. Elevated MVD is strongly correlated with metastatic potential and poor prognosis in invasive breast cancer [9], highlighting the critical need for precise evaluation of the tumor microvasculature for early diagnosis and prognostic stratification.

Conventional B-mode US assesses lesions based on echogenicity, margin, and posterior features according to the Breast Imaging Reporting and Data System (BI-RADS). Nevertheless, its diagnostic sensitivity is constrained by the frequent overlap in imaging features between benign and malignant lesions. Although Color Doppler Flow Imaging (CDFI) and Contrast-Enhanced US (CEUS) provide supplementary hemodynamic information, their ability to delineate microvasculature is limited. CDFI can detect vessels with diameters ≥200 μm but fails to visualize microvessels below 0.1 mm [10,11]. CEUS improves macroscopic perfusion assessment but still lacks the resolution for detailed microvascular analysis due to the physical diffraction limit of ultrasound.

Ultrasound Resolution Microscopy (URM), also referred to as Super-Resolution Ultrasound in some literature, is an emerging innovative technology that overcomes the traditional diffraction limit [12]. By precisely localizing and tracking individual microbubbles within the vasculature, URM achieves super-resolution imaging at the micrometer scale (typically 10–20 μm), enhancing resolution by an order of magnitude compared with conventional Doppler techniques [13,14]. This capability enables non-invasive, detailed depiction of microvascular morphology, density, and hemodynamics. URM has been successfully applied in preclinical models and clinical studies across various fields, including neurology, nephrology, and oncology, for visualizing microvasculature in the brain, kidneys, breast tumors, and lymph nodes [15,16,17,18,19]. Furthermore, quantitative parameters derived from URM, such as MVD, vascular complexity, and tortuosity, have shown promise in monitoring response to anti-angiogenic therapy [20]. Recent consensus statements on super-resolution ultrasound highlight its transformative potential in microvasculature imaging but underscore the need for robust, prospective clinical validation to establish diagnostic thresholds and clinical utility [12].

Despite its potential, the clinical value of URM in the differential diagnosis of breast lesions requires further validation. Therefore, this study aimed to prospectively evaluate the feasibility and diagnostic performance of quantitative URM parameters in distinguishing benign from malignant breast masses. Specifically, this work seeks to characterize microvascular features to lay the groundwork for future multi-center studies establishing standardized criteria, and to assess the incremental value of integrating URM with conventional BI-RADS.

## 2. Materials and Methods

### 2.1. Study Design and Patient Enrollment

This prospective, single-center pilot study was designed to assess the preliminary feasibility and effect sizes of URM. It was conducted at the Department of Ultrasound, the Second Affiliated Hospital of Anhui Medical University, between September 2024 and October 2025. The study protocol was approved by the Institutional Ethics Committee, and written informed consent was obtained from all participants.

A total of 55 female patients with 57 breast lesions were enrolled. All patients underwent B-mode ultrasound, color Doppler flow imaging (CDFI), contrast-enhanced ultrasound (CEUS), and ultrasound resolution microscopy (URM). Histopathological diagnosis was obtained via core-needle biopsy or surgical excision and served as the reference standard. Histopathological evaluations of all specimens were independently performed by two senior pathologists who were blinded to the patients’ clinical information and prior ultrasound/URM imaging findings. Any diagnostic discrepancies were resolved by consensus through consultation with the director of the pathology department.

**Figure 1:**
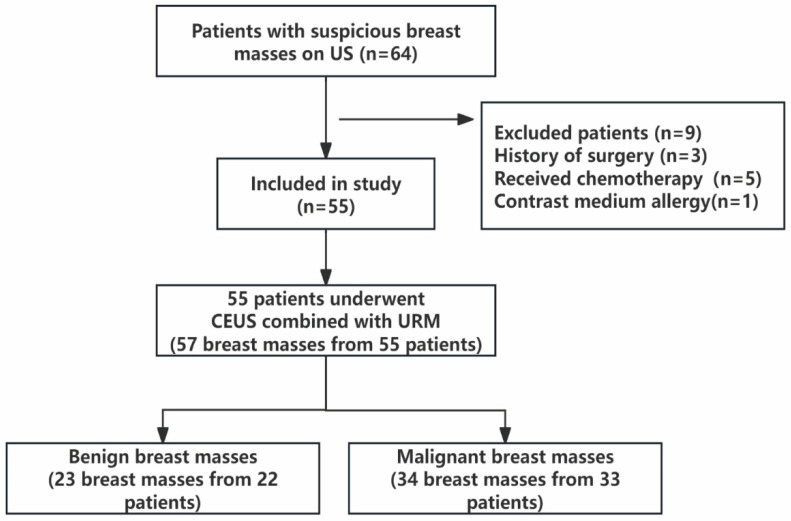
Study flow chart. CEUS = contrast-enhanced ultrasound, URM = Ultrasound Resolution Microscopy.

Inclusion criteria: (1) Women ≥18 years; (2) planned biopsy or surgery without contraindications; and (3) available and analyzable imaging data. Exclusion criteria: (1) Contrast allergy; (2) pregnancy or lactation; (3) cognitive or psychiatric disorders affecting compliance; and (4) a history of any prior treatment (e.g., surgery, radiotherapy, chemotherapy, or ablation) directed at the index breast lesion. The patient selection flowchart is presented in Figure 1.

### 2.2. Image Acquisition Protocol

All grey-scale, colour-Doppler and contrast-enhanced data were acquired on a VINNO Ultimus 9E system (VINNO Technology, Suzhou, China) with a 5–14 MHz linear probe by one breast-imaging radiologist (X.X., >15 years of experience) using fixed preset parameters to ensure uniform image quality. A 20-G intravenous cannula was placed in an antecubital vein. SonoVue (Bracco, Milan, Italy) was reconstituted with 5 mL of 0.9% saline; 2.5 mL of the suspension was injected as a rapid bolus, followed by 5 mL saline flush. With the probe fixed at the plane displaying the richest colour-Doppler signal within the lesion and the patient breathing quietly, native B-mode features were stored. During the subsequent contrast phase, three consecutive 10-s raw RF sequences (frame rate: 100 Hz) were acquired at the time points of agent arrival, peak enhancement and late wash-out, yielding 15 s of data for subsequent URM analysis.

### 2.3. URM Image Analysis

Two breast-imaging radiologists (X.F.L. with 15 years of experience and J.W. with 8 years) who were blinded to clinical and pathological data independently hand-drew the ROI along the hypoechoic rim on the B-mode frame matching the first URM sequence. Inter-reader overlap ≥95% was required; if it was lower, a third senior radiologist (X.X., >15 years) arbitrated by majority rule. After contour locking, the built-in software (URM QAT, VINNO Ultimus 9E; VINNO Technology, Suzhou, China) generated density and velocity maps.

**Figure 2:**
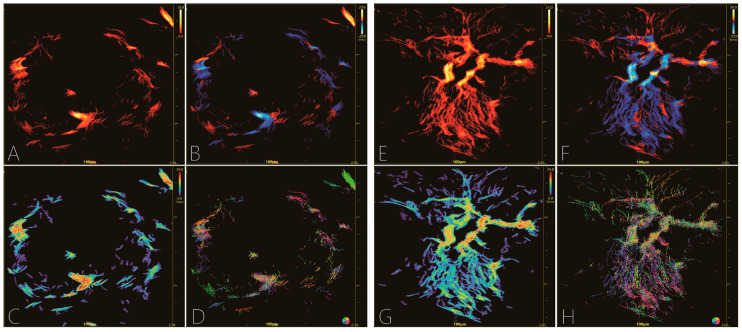
Representative URM images of pathologically confirmed breast lesions. Comparing the two, (**A**–**D**) Fibroadenoma demonstrates a hypovascular pattern with regular, smooth, and orderly vessel trajectories. Specifically, (**E**–**H**) Invasive breast carcinoma (NST) displays a hypervascular pattern with chaotic, tortuous, and distorted microvascular architecture. In contrast, (**A**,**E**) represent microvessel density maps, (**B**,**F**) flow-direction maps, (**C**,**G**) flow-velocity maps, and (**D**,**H**) flow-angle maps. Scale bars represent 100 µm, right-axis values indicate imaging depth (cm), and color bars show parameter-specific ranges optimized per case.

Exported indices: (1) Morphological indices: Mass Density (Max, Min, Mean); Vessel Ratio (%); ROI Max Curvature (DM = A/E, where A = actual mid-line vessel length and E = Euclidean end-to-end distance; DM = 1 implies a perfectly straight course), representing the maximal tortuosity of vessels within the ROI; Complexity Level (box-count fractal dimension estimating the rate of detail change versus scale change—a higher slope indicates more geometric disorder); and Vessel Count (mean number of vessels along the two longest perpendicular diameters). (2) Kinetic–Mass Velocity (Max, Min, Mean) and Blood Volume (BV, total micro-bubble count entering the ROI throughout the entire acquisition, expressed in arbitrary units). (3) Composite–Perfusion Index (Mean Vel × Vessel Ratio) and Entropy (Shannon entropy of density histogram = Density Entropy, of velocity histogram = Velocity Entropy; higher values denote greater irregularity). Representative URM images of benign and malignant lesions are compared in Figure 2.

### 2.4. Statistical Analysis

All statistical analyses were performed using SPSS (version 29.0; IBM Corp., Armonk, NY, USA) and R software (version 4.3.2; R Foundation for Statistical Computing, Vienna, Austria). Continuous variables were assessed for normality using the Shapiro–Wilk test. Normally distributed data are expressed as mean ± standard deviation and compared using the independent samples t-test; non-normally distributed data are presented as median (interquartile range) and analyzed using the Mann–Whitney U test. Categorical variables are expressed as frequencies and percentages and compared using the Pearson χ^2^ test or Fisher’s exact test, as appropriate. To strictly align with clinical biopsy thresholds, BI-RADS categories were dichotomized into benign (≤3) and suspicious/malignant (≥4A). To mitigate overfitting given the limited sample size and ensure an events-per-variable (EPV) ratio > 10, the 14 standardized (Z-score) URM parameters underwent 10-fold cross-validated least absolute shrinkage and selection operator (LASSO) regression. The top two URM features with the highest absolute standardized importance at the optimal λ (minimizing log-loss) were selected for further modeling.

Three logistic regression models were constructed: Model 1 (BI-RADS only), Model 2 (the two selected URM features), and Model 3 (Combined). To address quasi-complete separation (zero malignant events in BI-RADS ≤ 3), the robust likelihood ratio test (LRT) replaced the conventional Wald test for reliable *p*-value estimation. Diagnostic performance was evaluated using ROC curves (AUC, sensitivity, specificity, PPV, NPV), with optimal cut-offs determined by Youden’s index. AUCs were compared via DeLong’s test. Decision curve analysis (DCA) assessed the net clinical benefit. Statistical significance was defined as a two-tailed *p* < 0.05.

## 3. Results

### 3.1. Clinical and US Features

This study analyzed 57 pathologically confirmed breast lesions from 55 women (median age: 46 years; range: 22–73). Detailed clinical and pathological staging for the 34 malignant lesions is summarized in Supplementary Table S2. The cohort included 23 (40.4%) benign and 34 (59.6%) malignant lesions (Figure 1). As summarized in Table 1, patients in the malignant group were significantly older (51.09 ± 11.51 vs. 40.65 ± 10.27 years, *p* = 0.001) and presented with larger lesion sizes (29.0 ± 11.0 vs. 17.9 ± 9.5 mm, *p* < 0.001) compared to the benign group. However, there was no significant difference in baseline BMI between the two cohorts (23.09 ± 3.40 vs. 22.36 ± 2.74 kg/m^2^, *p* = 0.285). Malignant masses demonstrated significantly higher rates of suspicious US characteristics: irregular shape (97.1% vs. 34.8%, *p* < 0.001), non-circumscribed margins (94.1% vs. 30.4%, *p* < 0.001), and microcalcifications (85.3% vs. 21.7%, *p* < 0.001). No significant differences were observed in laterality, orientation, echo pattern, or macrocalcifications (all *p* > 0.05).

### 3.2. Histopathological Findings

The histology results are summarised in Table 2. Among the 23 benign lesions, mammary adenosis predominated (*n* = 10, 43.5%), followed by fibroadenoma (*n* = 7, 30.4%). The remaining seven benign cases comprised three intraductal papillomas, two foci of mammary-gland hyperplasia and one granulomatous mastitis. Of the 34 malignant lesions, 25 (73.5%) were invasive carcinoma of no special type (NST), six (17.6%) were ductal carcinoma in situ (DCIS) and two (5.9%) were invasive lobular carcinoma; one lesion proved to be mucinous carcinoma. Carcinomas of no special type accounted for the large majority of the invasive lesions, reflecting the spectrum ordinarily encountered in clinical practice.

### 3.3. Comparison of URM Quantitative Parameters

**Figure 3:**
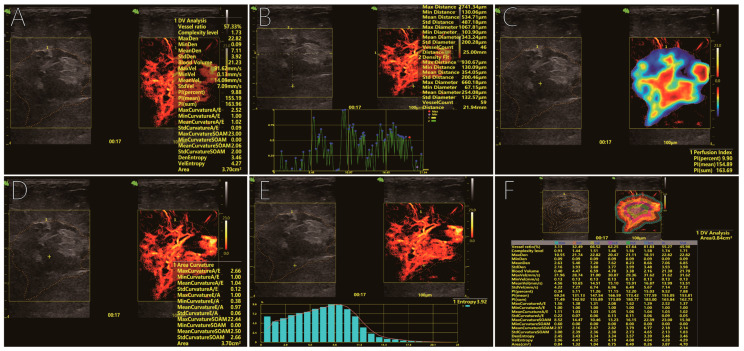
Quantitative analysis of URM parameters generated by manual segmentation. (**A**) Density–velocity map quantification. (**B**–**E**) Quantitative metrics for vessel count, perfusion index, ROI curvature and entropy, respectively. (**F**) Summary of rim-region parameters. Scale bars represent 100 µm, right-axis values indicate imaging depth (cm), and color bars show parameter-specific ranges optimized per case. Additionally, the red line overlaid on the histogram (e.g., in (**E**)) represents the fitted distribution curve of the corresponding quantitative parameter.

Table 3 summarises the ultrasound resolution microscopy (URM)-derived metrics. Aligned with URM’s dual-dimensional output of density and velocity, malignant masses showed pronounced alterations along both axes. Structurally, vessel ratio [23.49 (8.80–34.61) vs. 7.27 (3.60–13.64), *p* = 0.002], complexity level [1.55 (1.36–1.63) vs. 1.34 (1.16–1.41), *p* < 0.001] and vessel count [36.50 (16.25–45.88) vs. 11.0 (4.00–20.00), *p* < 0.001] were all elevated, accompanied by greater ROI maximum curvature [1.75 (1.39–2.03) vs. 1.29 (1.19–1.40), *p* < 0.001]. Functionally, mean velocity (8.84 ± 2.99 vs. 6.46 ± 2.66 cm s^−1^, *p* = 0.003), blood volume [6.52 (1.49–13.24) vs. 1.18 (0.54–1.82), *p* < 0.001] and perfusion index [3.11 (1.25–5.23) vs. 0.92 (0.36–1.49), *p* < 0.001] were significantly higher, whereas density extrema, velocity extrema and entropy metrics did not differ (all *p* > 0.05). The workflow for extracting and analyzing these quantitative parameters from URM images is depicted in Figure 3.

### 3.4. Feature Selection and Predictive Modeling

**Figure 4:**
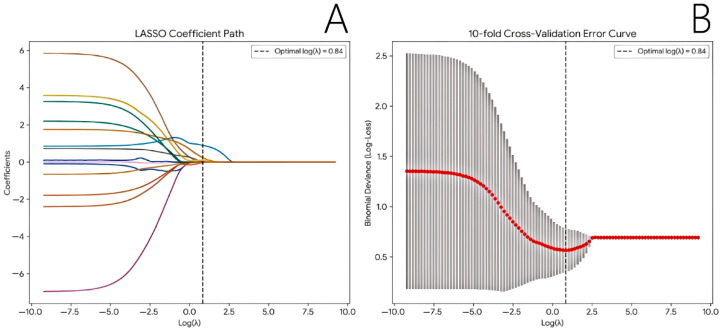
Feature selection using LASSO regression. (**A**) LASSO coefficient profiles of the 14 standardized quantitative ultrasound resolution microscopy (URM) parameters. Each colored solid line represents the coefficient path of an individual parameter. The vertical dashed line indicates the optimal penalization parameter (λ) determined by the 10-fold cross-validation. (**B**) The 10-fold cross-validation error curve. The binomial deviance (log-loss) is plotted against log(λ). The red dots represent the average deviance for each model, and the gray bars indicate the standard errors. The vertical dashed line identifies the optimal λ (log(λ) = 0.84) that minimizes the cross-validation error, which was subsequently used to select the core predictive features (VS and Max_Curvature) with non-zero coefficients.

To avoid overfitting and ensure a robust events-per-variable (EPV) ratio, the 14 standardized quantitative URM parameters were input into a LASSO regression model with 10-fold cross-validation. At the optimal penalization threshold (λ = 2.310, minimizing the binomial deviance), the coefficients of most parameters shrunk to zero, leaving exactly five features with non-zero coefficients (Figure 4). Among them, the top two features with the highest absolute standardized importance—VS and Max_Curvature—were identified as the core predictive variables and selected for subsequent modeling (Supplementary Table S1).

Based on these selected variables and the dichotomized BI-RADS categories (≤3 vs. ≥4A), three distinct logistic regression models were constructed: Model 1 (BI-RADS only), Model 2 (URM only), and Model 3 (Combined model). The detailed regression coefficients, odds ratios, and corresponding P-values are summarized in Table 4. Notably, due to the complete absence of malignant events in the benign BI-RADS subgroup, a quasi-complete separation occurred; thus, the robust likelihood ratio test was applied, confirming that BI-RADS remains a highly significant predictor (*p* < 0.001).

### 3.5. Diagnostic Performance and Clinical Utility

**Figure 5:**
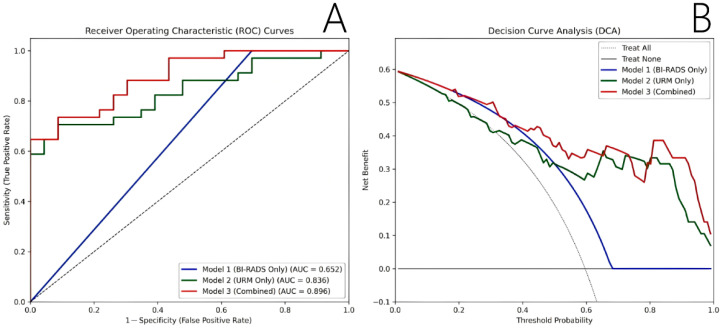
Diagnostic performance and clinical utility of the predictive models. (**A**) Receiver operating characteristic (ROC) curves for Model 1 (BI-RADS Only), Model 2 (URM Only), and Model 3 (Combined). The Combined model achieved the highest discriminative ability with an area under the curve (AUC) of 0.896, significantly outperforming the single-modality models. The black dashed line represents the reference line of no discrimination (AUC = 0.5). (**B**) Decision curve analysis (DCA) of the three models. The *y*-axis represents the net benefit, and the *x*-axis represents the threshold probability. Compared with the single-modality models and the default strategies of treating all patients (Treat All, gray dotted line) or treating no patients (Treat None, black solid line), the Combined model (red solid line) demonstrated the highest clinical net benefit across the majority of clinically relevant threshold probabilities.

The diagnostic performances of the three models are compared in Table 5 and Figure 5A. The Combined model (Model 3) achieved the highest discriminative ability, with an AUC of 0.896 (95% CI: 0.809–0.960), outperforming both the BI-RADS-only model (AUC = 0.652) and the URM-only model (AUC = 0.836). Crucially, while the BI-RADS-only model yielded 100% sensitivity but extremely low specificity (30.43%), the Combined model successfully maintained a high sensitivity of 73.53% while remarkably improving the specificity to 91.30%.

Finally, decision curve analysis (DCA) demonstrated the clinical utility of the models (Figure 5B). Across the majority of the clinically relevant threshold probabilities, the Combined model consistently provided the highest net benefit compared with the single-modality models and the “treat-all” or “treat-none” strategies, highlighting its superior value in clinical decision-making.

## 4. Discussion

In this prospective study, we evaluated the diagnostic utility of quantitative parameters derived from super-resolution ultrasound (URM) in differentiating benign and malignant breast lesions. To ensure statistical robustness and mitigate overfitting, we employed LASSO regression with cross-validation to distill 14 quantitative parameters down to the two most critical independent predictors: Vessel Count and Max_Curvature. Most importantly, our findings demonstrated that the Combined model—integrating these two URM microvascular features with the conventional BI-RADS category—yielded excellent diagnostic performance (AUC = 0.896), significantly outperforming the single-modality models and providing a superior net clinical benefit. Therefore, our study confirms and extends prior findings on microvascular differences in breast lesions using URM [19,21,22]. Its novel contribution lies in the development and clinical utility assessment of this combined quantitative URM and BI-RADS model, demonstrating a significant step towards practical clinical integration.

The quantitative URM assessment revealed a distinct vascular signature in malignant lesions, characterized by significantly elevated parameters such as vessel count, microvessel density, vascular curvature, complexity, average velocity, blood volume, and perfusion index. These findings collectively indicate a microvascular phenotype characterized by high vascular density and aberrant hemodynamics, which is a hallmark of tumor angiogenesis [17,23]. The increased vessel count and density reflect the prolific but dysregulated endothelial cell proliferation and sprouting driven by an imbalance between pro-angiogenic factors (e.g., VEGF, FGF) and their inhibitors. This process results in a chaotic, immature vascular architecture, which is quantitatively captured by URM as high vascular curvature and complexity [7,8,24]. The concomitant elevations in average velocity, blood volume, and perfusion index are hemodynamic consequences of this abnormal vasculature, which often contains arteriovenous shunts and lacks normal regulatory mechanisms, ultimately serving to meet the heightened metabolic demands of rapidly proliferating tumor cells [25,26].

To address the multicollinearity among the numerous significant URM parameters, we employed LASSO regression with cross-validation, which distilled the comprehensive microvascular profile down to two core independent predictors: Vessel Count and Max_Curvature. This finding should not be interpreted as indicating a lack of value for other significant parameters (such as perfusion index or complexity level), but rather that their diagnostic information is biologically and statistically interrelated with VS and curvature [23,27]. The inclusion of these representative parameters effectively encapsulates the hallmarks of tumor angiogenesis, providing a more objective basis for differentiation. This confirms that quantitative microvascular analysis effectively compensates for the limitations of traditional morphological assessment, consistent with previous studies highlighting the value of super-resolution microvascular imaging in improving diagnostic capability [21,28,29].

Building on this foundation, the Combined model, which integrated these quantitative URM features with the conventional BI-RADS category, achieved a robust diagnostic accuracy (AUC = 0.896), significantly outperforming the BI-RADS-alone approach (AUC = 0.652). Decision curve analysis (DCA) further confirmed that the Combined model provided the highest net benefit across a wide threshold probability range, demonstrating excellent clinical applicability.

The superiority of the Combined model stems from the complementary nature of the information provided: the conventional BI-RADS delineates macroscopic morphological characteristics (e.g., margins, calcifications), while URM reveals functional and microarchitectural features (e.g., microvascular abundance and tortuosity). The integration of these multidimensional data enables a more comprehensive and accurate lesion assessment, thereby offering greater utility for clinical decision-making. This synergistic effect is of paramount clinical importance. By drastically boosting specificity to 91.30% (compared to 30.43% in the BI-RADS-alone model) while maintaining an adequate sensitivity (73.53%), the Combined model directly addresses the unmet clinical need of reducing false positives. The significantly improved specificity of our combined approach perfectly aligns with the strategic goal of modern breast imaging guidelines to minimize unnecessary invasive biopsies while ensuring oncological safety [30,31], thereby alleviating patient anxiety and reducing healthcare costs.

Furthermore, the practical significance of the data obtained in this study is twofold, encompassing both pathophysiological insights and direct clinical utility. Pathophysiologically, as an emerging and highly sensitive non-invasive technology, URM provides a macroscopic quantitative window into the microscopic hallmarks of tumor neoangiogenesis [12]. Our comprehensive initial analysis revealed that malignant lesions consistently exhibit significantly elevated vessel ratios, perfusion indices, and complexity levels, reflecting the hyperactive angiogenic environment and chaotic vascular networks typical of breast cancer. To eliminate redundancy and identify the most robust imaging biomarkers, our LASSO regression distilled these comprehensive parameters down to two core independent predictors: Vessel Count and Max_Curvature. Specifically, Vessel Count directly quantifies the absolute abundance of microvessels, correlating strongly with the hypervascular nature and high microvascular density of malignancies. Concurrently, Max_Curvature captures the tortuous, highly irregular, and intertwined morphological architecture of malignant microvascular networks (e.g., arteriovenous shunts) [32].

Clinically, these quantitative pathophysiological insights translate into a powerful triage tool that addresses a major clinical dilemma. In routine practice, while conventional BI-RADS categorization yields high diagnostic sensitivity, its notoriously low specificity (frequently leading to false positives in the BI-RADS 4A subcategory) results in a high number of benign lesions being subjected to unnecessary core-needle biopsies [33]. Our data explicitly highlight this issue: the BI-RADS-alone model achieved an extremely low specificity of 30.43%. However, by integrating the core URM microvascular data with conventional BI-RADS, our Combined model significantly improved the overall diagnostic efficacy (AUC = 0.896) and drastically boosted specificity to 91.30%. This indicates that URM can serve as a highly effective “downgrading” strategy. For breast masses morphologically classified as suspicious (e.g., BI-RADS 4A) but demonstrating low-risk microvascular profiles on URM (e.g., low Vessel Count and lower curvature), clinicians might safely downgrade the assessment to recommend short-term follow-up rather than immediate biopsy, thereby sparing patients from invasive trauma and alleviating healthcare burdens without compromising oncological safety.

It is also noteworthy that microvascular imaging faces inherent challenges in differentiating hypervascular inflammatory conditions, such as granulomatous mastitis, which often mimics malignancy due to significant angiogenesis and hyperemia [19,34]. Consistent with this pathophysiology, inflammatory lesions in our cohort could exhibit an elevated Vessel Count on URM due to inflammatory vasodilation. However, while inflammation significantly increases microvessel density, it typically lacks the highly tortuous, chaotic, and irregular microvascular architecture—captured by Max_Curvature—that uniquely characterizes tumor neoangiogenesis. This physiological nuance perfectly underscores the rationale for our Combined model. Consequently, combining URM functional parameters (vascular abundance and tortuosity) with BI-RADS morphological assessments provides a more comprehensive evaluation. This combined approach effectively filters out false-positive cases caused by inflammatory lesions, which is the primary reason our combined model achieved a significantly improved specificity of 91.30%.

Our study has several limitations that merit consideration. First, as a prospective pilot study conducted at a single tertiary university-affiliated hospital, a design that may introduce selection bias, the generalizability of our findings to broader populations and clinical settings may be limited. Inherently restricted by the stringent imaging protocols of URM (e.g., intravenous contrast administration and absolute patient immobility), the sample size of 57 lesions remains modest. However, this cohort size is highly comparable to recent early-phase URM feasibility studies in breast imaging [19,21]. Furthermore, a post hoc power analysis revealed that our sample provides greater than 90% statistical power to detect the robust diagnostic efficacy (AUC = 0.896) of the combined model. While we rigorously mitigated the risk of model overfitting by employing LASSO dimensionality reduction and maintaining an events-per-variable (EPV) ratio > 10, the relatively small sample size still limits the capacity for extensive subgroup analyses. Additionally, although the papillary lesions in our cohort were pathologically benign, their recognized malignant potential warrants cautious interpretation and dedicated analysis in larger future studies.

A key limitation is the absence of a head-to-head correlation with dynamic contrast-enhanced MRI (DCE-MRI), the current clinical imaging standard for assessing tumor vasculature. In routine clinical practice, patients presenting with highly suspicious masses (e.g., BI-RADS ≥ 4A) on conventional ultrasound typically proceed directly to ultrasound-guided biopsy due to the cost and scheduling constraints of MRI, making paired MRI data unavailable for all participants. Nevertheless, the lack of this comparison represents a missed opportunity to precisely define the potential additive role of URM within the existing diagnostic pathway, especially for BI-RADS 4 lesions where MRI is frequently utilized according to current clinical guidelines [31]. Finally, although ROC analysis identified optimal cutoffs within this dataset, these values require external validation. Therefore, future large-scale, multicenter prospective studies are essential to confirm generalizability, establish robust and universally applicable diagnostic criteria, and directly compare URM with DCE-MRI to delineate their complementary roles. This strictly aligns with the recommended pathway for developing reliable quantitative imaging biomarkers [35].

## 5. Conclusions

In conclusion, quantitative URM imaging effectively captures the distinct microvascular heterogeneity of breast cancer, with features such as Vessel Count and Max_Curvature serving as robust independent predictors of malignancy. The integration of these core URM parameters with conventional BI-RADS categorization significantly enhances overall diagnostic accuracy (AUC = 0.896) and drastically improves specificity (91.30%). Consequently, URM demonstrates immense potential as a non-invasive, complementary diagnostic tool capable of optimizing clinical triage, safely downgrading equivocal lesions, and ultimately reducing the burden of unnecessary biopsies in routine clinical practice.

While these pilot results are promising, the translation of URM into clinical practice mandates standardized protocols and validation in large, prospective multicenter cohorts, as emphasized in recent field-wide consensus recommendations [12].

## Figures and Tables

**Table 1:** Baseline Clinical and Conventional US Characteristics.

Parameter	Benign (*n* = 23)	Malignant (*n* = 34)	*p*
Age (years)	40.7 ± 10.3	51.1 ± 11.5	0.001
BMI (kg/m^2^)	22.4 ± 2.7	23.1± 3.4	0.285
Size (mm)	17.9 ± 9.5	29.0 ± 11.0	<0.001
Position			0.471
Right	10 (43.5)	19 (55.9)	
Left	13 (56.5)	15 (44.1)	
Shape			<0.001
Oval, round	15 (65.2)	1 (2.9)	
Irregular	8 (34.8)	33 (97.1)	
Orientation			0.173
Parallel	19 (82.6)	27 (79.4)	
Not parallel	4 (17.4)	7 (20.6)	
Margin			<0.001
Smooth	16 (69.6)	2 (5.9)	
Irregular, angular, spiculate	7 (30.4)	32 (94.1)	
Echo pattern			0.731
Hyperechoic, isoechoic	4 (17.4)	4 (11.8)	
Hypoechoic	19 (82.6)	30 (88.2)	
Calcification			0.231
Absent	18 (78.3)	5 (14.7)	
Microcalcification	5 (21.7)	29 (85.3)	
BI-RADS Category			
≤3 (Benign)	7 (30.4)	0 (0.0)	<0.001
≥4A (Suspicious)	16 (69.6)	34 (100.0)	

Note: Continuous variables are presented as mean ± SD. Categorical variables are expressed as n (%). *p*-values were calculated with independent-sample t test for continuous variables and χ^2^ test for categorical variables. The comparison of echo patterns (hyperechoic/isoechoic vs. hypoechoic) was performed with Fisher’s exact test because the expected cell count was <5; the exact two-tailed *p* value is 0.731. *p* values indicate statistical significance (*p* < 0.05).

**Table 2:** Histologic Diagnoses.

Histologic Diagnosis	Masses (n%)
Benign lesions (*n* = 23)	
Mammary adenosis	10 (43.5)
Fibroadenoma	7 (30.4)
Intraductal papilloma	3 (13.0)
Granulomatous mastitis	1 (4.3)
Hyperplasia of the mammary glands	2 (8.7)
Malignant lesions (*n* = 34)	
Invasive carcinoma	25 (73.5)
Ductal carcinoma in situ	6 (17.6)
Invasive lobular carcinoma	2 (5.9)
Mucinous carcinoma	1 (2.9)

Note: Percentages are calculated within each diagnostic group (benign or malignant).

**Table 3:** Comparison of URM Parameters between Benign and Malignant Masses.

Parameter	Benign (*n* = 23)	Malignant (*n* = 34)	*p*-Value
Mass Density			
Max Den (mm)	21.00 (12.00, 26.00)	23.54 (20.43, 26.92)	0.167
Min Den (mm)	0.10 (0.07, 0.12)	0.10 (0.08, 0.11)	--
Mean Den (mm)	4.70 ± 2.29	5.80 ± 2.07	0.063
Vessel ratio (%)	7.27 (3.60, 13.64)	23.49 (8.80, 34.61)	**0.002**
Complexity level	1.34 (1.16, 1.41)	1.55 (1.36, 1.63)	**<0.001**
Vessel Count	11.00 (4.00, 20.00)	36.50 (16.25, 45.88)	**<0.001**
Max Curvature	1.29 (1.19, 1.40)	1.75 (1.39, 2.03)	**<0.001**
Mass Velocity			
Max Vel (mm/s)	28.00 (24.77, 29.77)	29.06 (25.95, 31.00)	0.113
Min Vel (mm/s)	0.12 (0.11, 0.12)	0.12 (0.11, 0.13)	0.515
Mean Vel (mm/s)	6.46 ± 2.66	8.84 ± 2.99	**0.003**
Blood Volume	1.18 (0.54, 1.82)	6.52 (1.49, 13.24)	**<0.001**
Perfusion Index	0.92 (0.36, 1.49)	3.11 (1.25, 5.23)	**<0.001**
Mass Entropy			
Density Entropy	3.37 (2.69, 3.76)	3.56 (3.31, 3.81)	0.162
Velocity Entropy	4.34 (4.13, 4.45)	4.32 (4.10, 4.40)	0.845

Note: Continuous data are presented as mean ± SD or median (IQR); comparisons using independent-sample *t* test or Mann–Whitney U test. Bold *p*-values indicate statistical significance (*p* < 0.05).

**Table 4:** Univariable and multivariable logistic regression analysis for predicting malignant lesions.

Model	Variable	β Coefficient	OR (95% CI)	*p*-Value
BI-RADS Only	BI_RADS	NE	NE	<0.001 *
URM Only	Vessel count	0.067	1.069 (1.014–1.127)	0.014
	Max_Curvature	1.604	4.973 (0.588–42.057)	0.141
Combined	BI_RADS	NE	NE	<0.001 *
	Vessel count	0.062	1.064 (0.998–1.134)	0.057
	Max_Curvature	2.618	13.713 (0.501–375.452)	0.121

Note: OR, odds ratio; CI, confidence interval; NE, not estimable. Due to the complete absence of malignant events in the subgroup with benign BI-RADS categories (≤3), a phenomenon of quasi-complete separation occurred in the data. Consequently, the conventional Wald test failed to provide reliable estimates for the regression coefficient and OR of the BI-RADS variable. *p*-values marked with an asterisk (*) were calculated using the robust likelihood ratio test (LRT) to overcome this separation issue.

**Table 5:** Diagnostic performance of different models for predicting malignant lesions.

Parameter	BI-RADS Only	URM Only	Combined
EPV	34.00	17.0	11.33
Cutoff	0.68	0.66	0.74
AUC (95% CI)	0.652 (0.560–0.750)	0.836 (0.730–0.932)	0.896 (0.809–0.960)
Sensitivity (%)	100.00	70.59	73.53
Specificity (%)	30.43	91.30	91.30
PPV (%)	68.00	92.31	92.59
NPV (%)	100.00	67.74	70.00
Accuracy (%)	71.93	78.95	80.70

Note: AUC, area under the curve; CI, confidence interval; PPV, positive predictive value; NPV, negative predictive value. The optimal cut-off values were determined by maximizing Youden’s index.

## Data Availability

The data presented in this study are available on request from the corresponding authors due to privacy restriction.

## Supplementary Materials

The following supporting information can be downloaded at: https://www.mdpi.com/article/10.3390/diagnostics16081119/s1, Table S1: Feature selection of quantitative URM parameters using LASSO regression; Table S2: Clinical/Pathological stages of the malignant breast lesions.

## Abbreviations

The following abbreviations are used in this manuscript:

URM	Ultrasound Resolution Microscopy
BI-RADS	Breast Imaging Reporting and Data System
ROC	Receiver Operating Characteristic
AUC	Area Under the Curve
DCA	Decision Curve Analysis
CEUS	Contrast-Enhanced Ultrasound
SMI	Superb Microvascular Imaging
ROI	Region of Interest
NST	Non-Special Type
DCIS	Ductal Carcinoma In Situ
MVD	Microvessel Density
