# Supplementary material for: Prospective Pilot Study of Ultrasound Resolution Microscopy Imaging (URM) for Differentiating Benign and Malignant Breast Lesions: A Quantitative Microvascular Parameter Analysis
Source: Diagnostics (Basel). 2026 Apr 8;16(8):1119. doi: 10.3390/diagnostics16081119 (PMC13114468; doi:10.3390/diagnostics16081119)
Supplement: Supplementary file 1 [file diagnostics-16-01119-s001.zip › diagnostics-4152054-supplementary.pdf]

Table S1: Feature selection of quantitative URM parameters using LASSO regression

**Rank**	**URM Features**	**LASSO Coefficient (Raw)**	**Standardized Importance**
**1**	**Vessel count**	**0.0535**	**0.9160**
**2**	**Max_Curvature**	**0.3608**	**0.2905**
3	Mean_Vel	0.0277	0.0844
4	Min_Vel	2.2598	0.0286
5	Blood_Volume	0.0108	0.0108

Note: Features are ranked in descending order based on their Standardized Importance (the absolute values of their standardized coefficients), which eliminates dimensional differences and represents their independent predictive contributions for identifying malignant lesions. The LASSO Coefficient (Raw) indicates the actual regression weight on the original scale of each feature. At the optimal penalization threshold (λ = 2.310), the coefficients of the remaining 9 URM features were shrunk to zero and thus excluded from this table. The top two ranked features (Vessel count and Max_Curvature) were subsequently selected to construct the final combined multivariable logistic regression model.

Table S2. Clinical/Pathological stages of the malignant breast lesions

Tumor Stage (n = 34)	n	%
Stage 0 (e.g., DCIS)	3	8.8
Stage I	3	8.8
Stage II	24	70.6
Stage III	4	11.8
Stage IV	0	0.0

*Note: Tumor staging was classified according to the 8th edition of the American Joint Committee on Cancer (AJCC) TNM staging system.
